# The Association of Low Admission Serum Creatinine with the Risk of Respiratory Failure Requiring Mechanical Ventilation: A Retrospective Cohort Study

**DOI:** 10.1038/s41598-019-55362-w

**Published:** 2019-12-10

**Authors:** Charat Thongprayoon, Wisit Cheungpasitporn, Api Chewcharat, Michael A. Mao, Sorkko Thirunavukkarasu, Kianoush B. Kashani

**Affiliations:** 1 Division of Nephrology and Hypertension, Department of Medicine, Mayo Clinic, Rochester, MN USA; 2 Division of Nephrology, Department of Internal Medicine, University of Mississippi Medical Center, Jackson, Mississippi USA; 3 Division of Nephrology and Hypertension, Mayo Clinic, Jacksonville, FL 32224 USA; 4 Division of Pulmonary and Critical Care Medicine, Department of Medicine, Mayo Clinic, Rochester, MN USA

**Keywords:** Outcomes research, Health care, Medical research, Nephrology, Risk factors

## Abstract

To assess the association between low serum creatinine (SCr) value at admission and the risk of respiratory failure requiring mechanical ventilation in hospitalized patients. A retrospective cohort study was conducted at a tertiary referral hospital. All hospitalized adult patients from 2011 through 2013 who had an admission SCr value were included in this study. Patients who were mechanically ventilated at the time of admission were excluded. Admission creatinine was stratified into 7 groups: ≤0.4, 0.5–0.6, 0.7–0.8, 0.9–1.0, 1.1–1.2, 1.3–1.4, and ≥1.5 mg/dL. The primary outcome was the occurrence of respiratory failure requiring mechanical ventilation during hospitalization. Logistic regression analysis was used to assess the independent risk of respiratory failure based on various admission SCr, using SCr of 0.7–0.8 mg/dL as the reference group in the analysis of all patients and female subgroup and of 0.9–1.0 mg/dL in analysis of male subgroup. A total of 67,045 eligible patients, with the mean admission SCr of 1.0 ± 0.4 mg/dL, were studied. Of these patients, 799 (1.1%) had admission SCr of ≤0.4 mg/dL, and 2886 (4.3%) developed respiratory failure requiring mechanical ventilation during hospitalization. The U-curve relationship between admission SCr and respiratory failure during hospitalization was observed, with the nadir incidence of in-hospital respiratory failure in SCr of 0.7–0.8 mg/dL and increased in-hospital respiratory failure associated with both reduced and elevated admission SCr. After adjustment for confounders, very low admission SCr of ≤0.4 mg/dL was significantly associated with increased in-hospital respiratory failure (OR 3.11; 95% CI 2.33–4.17), exceeding the risk related to markedly elevated admission SCr of ≥1.5 mg/dL (OR 1.61; 95% CI 1.39–1.85). The association remained significant in the subgroup analysis of male and female patients. Low SCr value at admission is independently associated with increased in-hospital respiratory failure requiring mechanical ventilation in hospitalized patients.

## Introduction

Creatinine is a breakdown metabolite of creatine phosphate in muscle^1^. It is eliminated by kidneys, mostly via glomerular filtration and partly via secretion at proximal tubule^1,2^. Therefore, creatinine clearance could be used to estimate the glomerular filtration rate (eGFR) by measuring serum creatinine (SCr), urine creatinine, and volume^3,4^. Additionally, SCr can be a reflection of muscle mass and diet. Some patients might have decreased SCr independent of eGFR such as vegetarians, patients with muscle wasting or critically ill individuals^3,5–7^.

Respiratory failure is a serious condition caused by either lung pathology resulting in imbalances in oxygen or carbon dioxide exchanges. Ventilation of air in the lungs closely depends on strength and workload of respiratory muscles^8^. Failure of ventilation is one of the major causes of acute respiratory failure. Management of respiratory failure includes treating the primary cause of respiratory failure and ventilatory support. The respiratory support could include nasal cannula, noninvasive ventilation, invasive ventilation, and ECMO^9^. In severe respiratory failure, to improve gas exchange and decrease work of breathing, mechanical ventilation is often needed^10^. Malnutrition, premorbid frailty, and severity of illness (measured by Acute Physiologic Assessment and Chronic Health Evaluation (APACHE) II score) are associated with a higher need of mechanical ventilation among respiratory failure patients. Even though low SCr is associated with poor nutritional status and decreased muscle mass, low SCr has not been reported in the literature to be a predictor of respiratory failure requiring mechanical ventilation.

Therefore, our goal is to investigate the association between low SCr at admission and the risk of respiratory failure requiring mechanical ventilation.

## Materials and Methods

### Study population

We conducted this retrospective cohort study at a tertiary referral center care. We explored our hospital database to collect data on all patients aged ≥18 years and admitted to Mayo Clinic, Minnesota, between January 1^st^, 2011 and December 31^st^, 2013, who had available SCr measurement at the time of admission. Patients who had end-stage renal disease or were on mechanical ventilation at hospital admission were excluded. ESRD was determined by *International Classification of Diseases*, 9th Revision, (ICD-9) diagnosis code of 458.21, 585.5, 585.6, 792.5, 996.56, 996.68, and 996.73 or by eGFR < 15 mL/min/1.73 m^2^. If a patient had recurring hospital admissions throughout the study period, we used only data on the first hospital admission. Mayo Clinic Institutional Review Board reviewed and approved this project (IRB number 15-000024) and waived informed consent due to the minimal risk nature of this study. The study was conducted in accordance with the relevant guidelines and regulations.

### Data collection

We collected the needed data from our institutional electronic medical record system. This database comprises demographic characteristics, diagnosis and procedure codes, hospital admission information, flow sheet information of inpatients and outpatients, and results of laboratory test. We defined the admission SCr as the initial SCr level in 24 hours of hospital admission. We categorized admission SCr into 7 groups: ≤0.4, 0.5–0.6, 0.7–0.8, 0.9–1.0, 1.1–1.2, 1.3–1.4 and ≥1.5 mg/dL. We calculated body mass index (BMI) using (weight in kg/[height in m]^2^) at admission. We grouped principal diagnoses based on ICD-9 code. We collected comorbid condition utilizing a previously validated information abstraction algorithm and calculated the Charlson Comorbidity Index (CCI)^11^ to evaluate comorbidity burden.

### Clinical outcomes

The outcome of interest was respiratory failure requiring mechanical ventilation during hospitalization. We did not include the elective use of mechanical ventilation during procedure or operation as the outcome. We also assessed the duration of mechanical ventilation among patients with RF requiring mechanical ventilation.

### Statistical analysis

We summarize continuous variables as mean ± standard deviation (SD) and categorical variables as number (%).We compared baseline demographics and clinical characteristics between admission SCr groups utilizing the χ^2^ test for categorical variables and analysis of variance (ANOVA) for continuous variables. The restricted cubic spline, with six knots were placed at 0.4, 0.6, 0.8, 1.0, 1.2 and 1.4 mg/dL of serum creatinine, was utilized to depict the potential non-linear association between SCr at the admission and risk of respiratory failure requiring mechanical ventilation. In the analysis of all patients, an admission SCr group of 0.7–0.8 mg/dL was used as the reference group for comparison. For the subgroup analyses, we use the same SCr range for female patients, whereas we selected an admission SCr group of 0.9–1.0 mg/dL in the analysis of male patients. These SCr limits were associated with the lowest unadjusted in-hospital mortality. We performed logistic regression analysis to assess the independent association of SCr levels with the development of RF requiring mechanical ventilation during hospitalization. We constructed a multivariable model to adjust for priori–defined covariates, including age, race, sex, BMI, principal diagnosis, CCI, diabetes mellitus, congestive heart failure, coronary artery disease, peripheral vascular disease, stroke, chronic obstructive pulmonary disease, hemiplegia/paraplegia, or cirrhosis. We performed subgroup analysis stratified by sex and tested interaction between sex and admission SCr on respiratory failure outcome. We performed linear regression analysis to assess the independent association between admission SCr levels and mechanical ventilation duration among patients with respiratory failure. As mechanical ventilation duration had a skewed distribution, hence, we summarized duration as median with interquartile range (IQR) and we log-transformed its value before entering into the model (Fig. S1). We reported the relative prolongation, which was derived from exponential of the regression coefficient. We considered a 2-tailed *P* value <0.05 statistically significant. When not specified, we performed all analyses using JMP statistical software Version 10 (SAS Institute Inc., USA). We constructed restricted cubic spline using STATA version 14.1 (StataCorp LLC, Texas, USA).

### Institution

This work was performed at Mayo Clinic in Rochester, MN.

## Results

### Baseline characteristics

Among 76,696 hospitalized patients with available SCr at hospital admission through the study period, we excluded 2,702 ESRD patients and 6,949 patients who were on mechanical ventilation at hospital admission to include a total of 67,045 patients in the analysis. Table 1 describes the clinical characteristics. The mean age was 61 ± 18 years. Fifty-two percent of the enrolled patients were men. The mean BMI and admission SCr were 29.8 ± 7.7 kg/m^2^ and 1.0 ± 0.4 mg/dL, respectively. Very low admission SCr of ≤0.4 mg/dL was observed in 779 (1.2%) patients, whereas 6,174 (9.2%) had markedly elevated admission SCr of ≥1.5 mg/dL. Patients with low admission SCr tended to be younger, female, non-White, with lower BMI, and albumin while they had a higher prevalence of hemiplegia/paraplegia. The association of various admission SCr levels with CCI revealed a U-shaped distribution, with a nadir of CCI at an admission SCr of 0.7–0.8 mg/dL (*P* < 0.001).

**Table 1:** Baseline clinical characteristics.

Variables	All	Serum creatinine level at hospital admission (mg/dL)								
		≤0.4	0.5–0.6	0.7–0.8	0.9–1.0	1.1–1.2	1.3–1.4	≥1.5	p	p-for trend
N	67045	799	9021	20422	17413	9122	4094	6174		
Age, year	61 ± 18	54 ± 18	55 ± 18	58 ± 18	61 ± 17	65 ± 17	69 ± 15	71 ± 15	<0.001	<0.001
Male	34722 (52)	139 (17)	1451 (16)	7703 (38)	11553 (66)	6674 (73)	2879 (70)	4323 (70)	<0.001	<0.001
Caucasian	62418 (93)	681 (85)	8150 (90)	19022 (93)	16320 (94)	8594 (94)	3852 (94)	5799 (94)	<0.001	<0.001
BMI, kg/m^2^	29.8 ± 7.7	25.8 ± 7.8	28.4 ± 8.1	29.5 ± 7.8	30.1 ± 7.4	30.3 ± 7.2	30.5 ± 7.5	30.8 ± 7.9	<0.001	<0.001
Principal Diagnosis									<0.001	—
Cardiovascular	11653 (17)	57 (7)	864 (10)	2769 (14)	3160 (18)	2068 (23)	1028 (25)	1707 (28)		
Endocrine/Metabolic	1851 (3)	38 (5)	286 (3)	571 (3)	358 (2)	199 (2)	130 (3)	269 (4)		
Gastrointestinal	6651 (10)	100 (13)	1068 (12)	2185 (11)	1556 (9)	766 (8)	387 (9)	589 (10)		
Hematology/Oncology	11138 (17)	118 (15)	1522 (17)	3208 (16)	3039 (17)	1646 (18)	709 (17)	896 (15)		
Infectious Disease	1935 (3)	26 (3)	234 (3)	495 (2)	384 (2)	260 (3)	160 (4)	376 (6)		
Respiratory	2670 (4)	80 (10)	346 (4)	753 (4)	623 (4)	353 (4)	194 (5)	321 (5)		
Injury/poisoning	10539 (16)	142 (18)	1514 (17)	3344 (16)	2852 (16)	1418 (16)	563 (14)	706 (11)		
Other	20608 (31)	127 (30)	3187 (35)	7097 (35)	5441 (31)	2412 (26)	923 (23)	1310 (21)		
Charlson score	1.7 ± 2.3	1.9 ± 2.5	1.5 ± 2.3	1.4 ± 2.1	1.5 ± 2.2	1.9 ± 2.3	2.5 ± 0.6	3.0 ± 2.7	<0.001	<0.001
Comorbidities										
CAD	4738 (7)	29 (4)	273 (3)	950 (5)	1210 (7)	809 (9)	530 (13)	937 (15)	<0.001	<0.001
CHF	4010 (6)	24 (3)	209 (2)	624 (3)	828 (5)	691 (8)	496 (12)	1138 (18)	<0.001	<0.001
PVD	1839 (3)	14 (2)	121 (1)	341 (2)	376 (2)	310 (3)	211 (5)	466 (8)	<0.001	<0.001
Stroke	4712 (7)	37 (5)	416 (5)	1076 (5)	1129 (6)	747 (8)	447 (11)	860 (14)	<0.001	<0.001
DM	12457 (19)	138 (17)	1297 (14)	2949 (14)	2824 (16)	1882 (21)	1107 (27)	2260 (37)	<0.001	<0.001
COPD	5456 (8)	80 (10)	616 (7)	1287 (6)	1198 (7)	882 (10)	484 (12)	909 (15)	<0.001	<0.001
Cirrhosis	1532 (2)	27 (3)	214 (2)	387 (2)	272 (2)	191 (2)	127 (3)	314 (5)	<0.001	<0.001
Hemi/paraplegia	317 (0.4)	25 (3)	102 (1)	83 (0.4)	42 (0.2)	24 (0.3)	17 (0.4)	24 (0.4)	<0.001	<0.001
Albumin. g/dL (n = 8733)	3.6 ± 0.7	3.1 ± 0.7	3.4 ± 0.7	3.6 ± 0.7	3.7 ± 0.7	3.6 ± 0.7	3.5 ± 0.7	3.4 ± 0.7	<0.001	<0.001

Continuous data are presented as mean ± SD; categorical data are presented as count (%).

Abbreviations: BMI, body mass index; CAD, coronary artery disease; CHF, congestive heart failure; COPD, chronic obstructive pulmonary disease; DM, diabetes mellitus; PVD, peripheral vascular disease;

### Respiratory failure requiring mechanical ventilation

**Figure 1:**
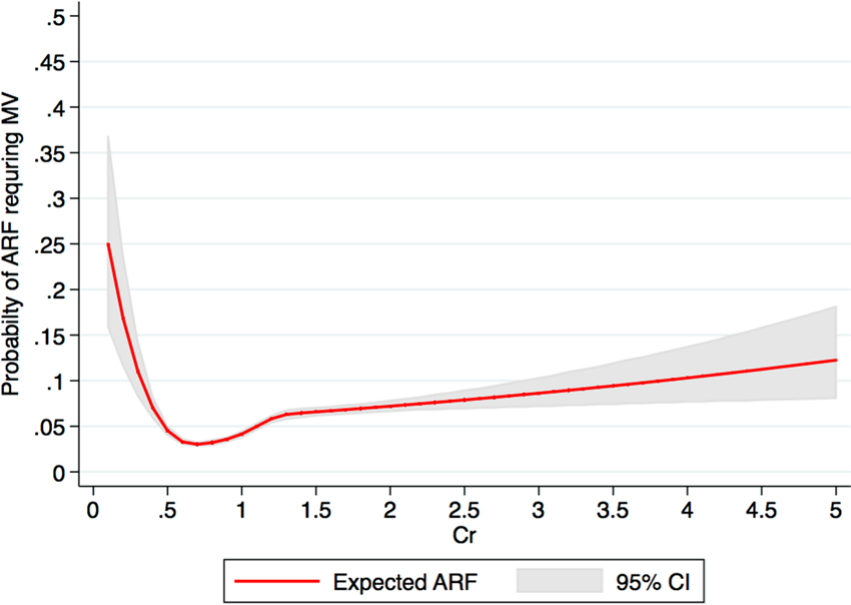
A restricted cubic spline showed the association between admission serum creatinine and risk of in-hospital respiratory failure requiring mechanical ventilation. Figure was created using STATA (StataCorp. 2015. Stata Statistical Software: Release 14. College Station, TX: StataCorp LP).

Among 67,045 patients, 2,886 (4.3%) developed respiratory failure requiring mechanical ventilation during hospitalization. We found a U-shaped distribution for in-hospital respiratory failure and admission SCr levels, with the lowest in-hospital respiratory failure found in patients with admission SCr of 0.7–0.8 mg/dL (Fig. 1). When adjusted for age, race, sex, BMI, principal diagnosis, and comorbidities, low admission SCr of ≤0.4, 0.5–0.6 mg/dL, and elevated admission of 1.1–1.2, 1.3–1.4, and ≥1.5 mg/dL were significantly associated with increased in-hospital respiratory failure, compared with the admission SCr of 0.7–0.8 mg/dL (Table 2). The risk associated with very low SCr of ≤0.4 mg/dL surpassing the risk associated with high SCr of ≥1.5 mg/dL (OR 3.11 vs. 1.61, respectively).

**Table 2:** The association between admission serum creatinine levels and in-hospital respiratory failure requiring mechanical ventilation.

Serum creatinine level at hospital admission (mg/dl)	Mechanical ventilator in hospital	Univariate analysis		Multivariate analysis	
		OR (95% CI)	p	Adjusted OR (95% CI)*	P
≤0.4	69 (8.6)	2.87 (2.22–3.72)	<0.01	3.11 (2.33–4.17)	<0.001
0.5–0.6	315 (3.5)	1.10 (0.96–1.26)	0.17	1.29 (1.11–1.50)	0.001
0.7–0.8	650 (3.2)	1 (ref)	—	1 (ref)	—
0.9–1.0	670 (3.9)	1.22 (1.09–1.36)	0.001	1.07 (0.95–1.21)	0.23
1.1–1.2	472 (5.2)	1.66 (1.47–1.87)	<0.001	1.30 (1.13–1.49)	0.001
1.3–1.4	250 (6.1)	1.98 (1.70–2.30)	<0.001	1.45 (1.23–1.71)	<0.001
≥1.5	460 (7.5)	2.45 (2.17–2.77)	<0.001	1.61 (1.39–1.85)	<0.001

*Adjusted for age, sex, race, BMI, principal diagnosis, Charlson Comorbidity Index, coronary artery disease, congestive heart failure, peripheral vascular disease, stroke, diabetes mellitus, chronic obstructive pulmonary disease, cirrhosis, hemi/paraplegia.

### Subgroup analysis of respiratory failure based on sex

**Figure 2:**
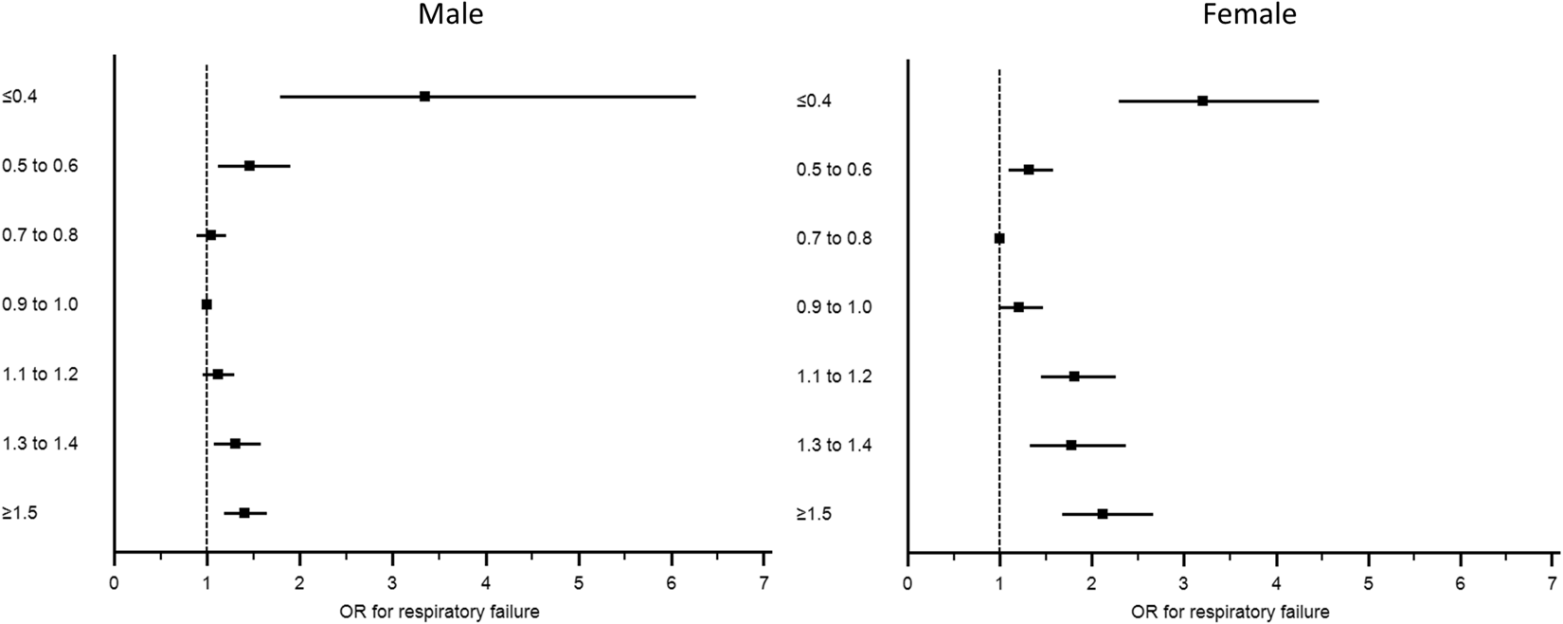
The association between admission serum creatinine and in-hospital respiratory failure requiring mechanical ventilation stratified by sex.

Among 34,722 male patients, the mean admission SCr level was 1.1 ± 0.4 mg/dL. Very low admission SCr of ≤0.4 noted in 139 (0.4%) and 1,737 (5.0%) developed respiratory failure requiring mechanical ventilation during hospitalization. The lowest incidence of in-hospital respiratory failure was in male patients with admission SCr of 0.9–1.0 mg/dL (4.1%). In adjusted analysis, low admission SCr of ≤0.4, 0.5–0.6 mg/dL, and elevated admission SCr of 1.3–1.4, and ≥1.5 mg/dL were significantly associated with higher risk of in-hospital respiratory failure compared with admission SCr of 0.9–1.0 mg/dL (Table 3a, Fig. 2).

**Table 3:** The association between admission serum creatinine levels and in-hospital respiratory failure requiring mechanical ventilation stratified by sex

(a)Male (n = 34722)					
Serum creatinine level at hospital admission (mg/dl)	Mechanical ventilator in hospital	Univariate analysis		Multivariate analysis	
		OR (95% CI)	p	Adjusted OR (95% CI)*	P
≤0.4	15 (10.8)	2.83 (1.64–4.87)	0.001	3.35 (1.79–6.26)	0.001
0.5–0.6	79 (5.4)	1.34 (1.05–1.72)	0.02	1.46 (1.12–1.90)	0.007
0.7–0.8	326 (4.2)	1.03 (0.89–1.19)	0.66	1.04 (0.89–1.21)	0.63
0.9–1.0	474 (4.1)	1 (ref)	—	1 (ref)	—
1.1–1.2	339 (5.1)	1.25 (1.08–1.44)	0.002	1.12 (0.96–1.30)	0.16
1.3–1.4	180 (6.3)	1.56 (1.31–1.86)	<0.001	1.31 (1.08–1.58)	0.006
≥1.5	324 (7.5)	1.89 (1.64–2.19)	<0.001	1.40 (1.19–1.65)	<0.001
**(b) Female (n = 32323)**					
≤0.4	54 (8.2)	3.41 (2.52–4.60)	<0.001	3.20 (2.29–4.47)	<0.001
0.5–0.6	236 (3.1)	1.23 (1.04–1.46)	0.02	1.32 (1.10–1.58)	0.003
0.7–0.8	324 (2.6)	1 (ref)	—	1 (ref)	—
0.9–1.0	196 (3.3)	1.32 (1.11–1.59)	0.003	1.21 (0.99–1.47)	0.06
1.1–1.2	133 (5.4)	2.20 (1.79–2.70)	<0.001	1.81 (1.45–2.26)	<0.001
1.3–1.4	70 (5.8)	2.34 (1.79–3.05)	<0.001	1.78 (1.33–2.37)	0.001
≥1.5	136 (7.4)	3.03 (2.47–3.73)	<0.001	2.12 (1.68–2.67)	<0.001

Adjusted for age, race, BMI, principal diagnosis, Charlson Comorbidity Index, coronary artery disease, congestive heart failure, peripheral vascular disease, stroke, diabetes mellitus, chronic obstructive pulmonary disease, cirrhosis, hemi/paraplegia.

P for interaction = 0.01.

Among 32,323 female patients, the mean admission SCr was 0.8 ± 0.3 mg/dL. Very low admission SCr ≤0.4 was seen in 660 (2.0%) patients, and 1,149 (3.6%) developed respiratory failure requiring mechanical ventilation during hospitalization. The lowest incidence of in-hospital respiratory failure was found in female patients with admission SCr of 0.7–0.8 mg/dL (2.6%). In adjusted analysis, low admission SCr of ≤0.4, 0.5–0.6 mg/dL, and elevated admission SCr of 1.1–1.2, 1.3–1.4, and ≥1.5 mg/dL were significantly associated with increased risk of in-hospital respiratory failure compared with admission SCr of 0.7–0.8 mg/dL (Table 3b, Fig. 2). There was an interaction between sex and admission SCr on the risk of in-hospital respiratory failure (p for interaction = 0.01).

### Duration of mechanical ventilation among respiratory failure patients

**Figure 3:**
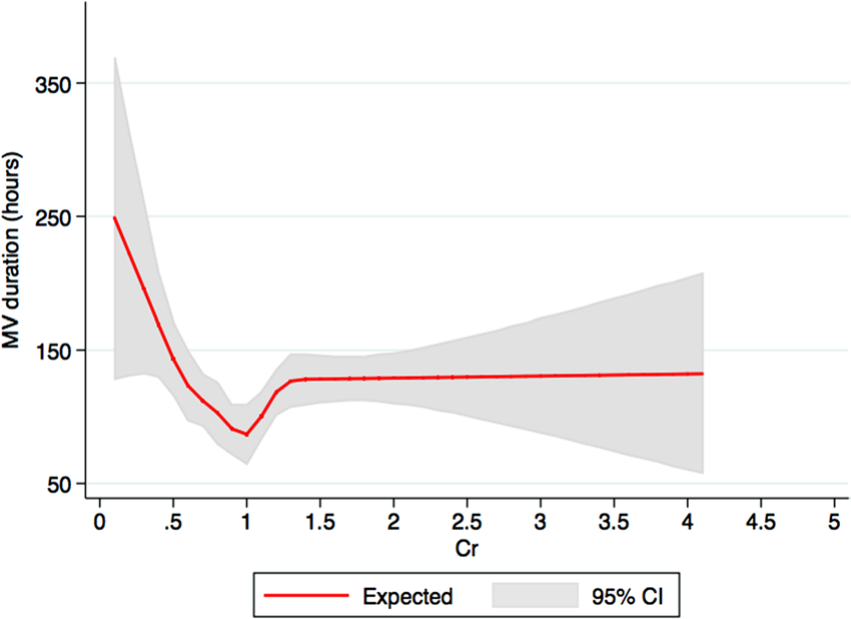
A restricted cubic spline showed the association between admission serum creatinine and the duration of mechanical ventilation among respiratory failure patients. Figure was created using STATA (StataCorp. 2015. Stata Statistical Software: Release 14. College Station, TX: StataCorp LP).

The median duration of mechanical ventilation was 46 (IQR 20-117) hours among patients with respiratory failure. A restricted cubic spline in Fig. 3 demonstrated a non-linear association between admission serum creatinine and duration of mechanical ventilation. In adjusted analysis, very low admission SCr was associated with 1.44-time longer duration of mechanical ventilation, whereas markedly elevated admission SCr was associated with 1.23-time longer duration of mechanical ventilation, compared with admission SCr of 0.7–0.8 mg/dL (Table 4). In subgroup analysis based on sex, there was no significant association between admission serum creatinine and duration of mechanical duration in male patients but low admission serum creatinine (≤0.6 mg/dL) was significantly associated with longer duration of mechanical ventilation in female patients (Table S1).

**Table 4:** The association between admission serum creatinine levels and duration of mechanical ventilation among respiratory failure patients.

Serum creatinine level at hospital admission (mg/dl)	Mechanical ventilation duration (hours)	Univariate analysis		Multivariate analysis	
		Relative prolongation (95% CI)	p	Relative prolongation (95% CI)*	P
≤0.4	79 (36–241)	1.70 (1.19–2.44)	0.004	1.44 (1.02–2.07)	0.04
0.5–0.6	56 (25–141)	1.28 (1.03–1.58)	0.03	1.25 (0.99–1.57)	0.06
0.7–0.8	41 (19–112)	1 (ref)	—	1 (ref)	—
0.9–1.0	41 (18–94)	0.89 (0.74–1.07)	0.20	0.97 (0.80–1.17)	0.75
1.1–1.2	44 (20–118)	1.04 (0.86–1.26)	0.66	1.16 (0.96–1.41)	0.13
1.3–1.4	51 (21–119)	1.06 (0.85–1.32)	0.62	1.17 (0.93–1.48)	0.19
≥1.5	51 (21–122)	1.18 (0.98–1.42)	0.08	1.23 (1.01–1.51)	0.04

Adjusted for age, sex, race, BMI, principal diagnosis, Charlson Comorbidity Index, coronary artery disease, congestive heart failure, peripheral vascular disease, stroke, diabetes mellitus, chronic obstructive pulmonary disease, cirrhosis, hemi/paraplegia.

## Discussion

Our findings revealed that either SCr ≤0.6 mg/dL or ≥1.1 mg/dL at admission was significantly associated with a higher risk of respiratory failure requiring mechanical ventilation. Low SCr at admission ≤0.6 mg/dL in both men and women was significantly associated with a higher risk of respiratory failure requiring mechanical ventilation. Furthermore, low SCr at admission ≤0.4 mg/dL was significantly associated with longer duration of mechanical ventilation compared to admission level of SCr 0.7–0.8 mg/dL.

To the best of our knowledge, we are the first group to report a lower SCr as an independent predictor for risk of respiratory failure requiring mechanical ventilation as well as an independent predictor for risk of prolonged duration of mechanical ventilation. We also depicted a U-curved association between SCr at admission and risk of respiratory failure requiring mechanical ventilation. Low SCr could reflect muscular wasting or sarcopenia and poor nutritional status^12,13^, which are strong risk factors for mechanical ventilation and a predictor of difficult weaning for mechanical ventilation as a reserved function of respiratory muscle and diaphragm among these patients is lesser than normal patients^3,13,14^. Moreover, malnutrition also results in weaker cellular mediated immunity and impaired surfactant production^15,16^. Even though volume overload could dilute SCr and result in lower levels^17,18^, we used SCr at admission before fluid resuscitation occurred and also adjusted for congestive heart failure to reduce the likelihood that fluid overload confounded the association. While high SCr could be due to acute kidney injury originated from the severity of primary illness, sepsis, or other causes^19,20^, all these conditions increase the risk of respiratory failure requiring mechanical ventilation as well.

Several limitations of this study are worth mentioning. This is a retrospective observational study so most likely there would be some residual confounders. Also, this is a single-center study with mostly White patients which may impact the generalizability of our data. Furthermore, we used only SCr at admission as a laboratory value without considering other laboratory values such as electrolytes or albumin, which might be potential confounders as well. However, there are several strengths to our study. We enrolled a large cohort of 67,045 patients to be able to adjust for several confounders which were specified a priori. Also, we used restricted cubic spline to visualize a potential non-linear association between SCr and risk of respiratory failure requiring mechanical ventilation. Last but not least, we also explored for effect modification by sex on this association.

In summary, low SCr level at admission is independently associated with increased in-hospital respiratory failure requiring mechanical ventilation. This association has been observed in patients of both sexes. Additionally, SCr ≤0.4 or ≥1.5 mg/dL were significantly associated with longer duration of mechanical ventilation compared to patients with SCr level at the admission of 0.7–0.8 mg/dL.

## Supplementary information

Supplementary information

## References

[1] Andrews R, Greenhaff P, Curtis S, Perry A, Cowley AJ (1998). The effect of dietary creatine supplementation on skeletal muscle metabolism in congestive heart failure. Eur Heart J.

[2] Thongprayoon C, Cheungpasitporn W, Kittanamongkolchai W, Harrison AM, Kashani K (2017). Prognostic Importance of Low Admission Serum Creatinine Concentration for Mortality in Hospitalized Patients. Am J Med.

[3] Park J (2013). Serum creatinine level, a surrogate of muscle mass, predicts mortality in peritoneal dialysis patients. Nephrol Dial Transplant.

[4] Schutte JE, Longhurst JC, Gaffney FA, Bastian BC, Blomqvist CG (1981). Total plasma creatinine: an accurate measure of total striated muscle mass. J Appl Physiol Respir Environ Exerc Physiol.

[5] Pasala S, Carmody JB (2017). How to use… serum creatinine, cystatin C and GFR. Archives of disease in childhood - Education &amp; practice edition.

[6] Thongprayoon C, Cheungpasitporn W, Kashani K (2016). Serum creatinine level, a surrogate of muscle mass, predicts mortality in critically ill patients. J Thorac Dis.

[7] Cartin-Ceba R, Afessa B, Gajic O (2007). Low baseline serum creatinine concentration predicts mortality in critically ill patients independent of body mass index. Crit Care Med.

[8] Scala R, Heunks L (2018). Highlights in acute respiratory failure. European Respiratory Review.

[9] Narendra DK (2017). Update in Management of Severe Hypoxemic Respiratory Failure. Chest.

[10] Gil A (1998). Influence of mechanical ventilation on blood lactate in patients with acute respiratory failure. Intensive Care Med.

[11] Charlson M, Szatrowski TP, Peterson J, Gold J (1994). Validation of a combined comorbidity index. J Clin Epidemiol.

[12] Assy N (2006). The changes in renal function after a single dose of intravenous furosemide in patients with compensated liver cirrhosis. BMC Gastroenterol.

[13] Thomas ME (2015). The definition of acute kidney injury and its use in practice. Kidney Int.

[14] Kou HW (2019). Sarcopenia is an effective predictor of difficult-to-wean and mortality among critically ill surgical patients. PLoS One.

[15] Law DK, Dudrick SJ, Abdou NI (1973). Immunocompetence of patients with protein-calorie malnutrition. The effects of nutritional repletion. Ann Intern Med.

[16] Garbagni R, Coppo F, Grassini G, Cardellino G (1968). Effects of lipide loading and fasting on pulmonary surfactant. Respiration.

[17] Lieu C, Anderson R (2007). Serum creatinine: why lower may not be better. Crit Care Med.

[18] Thongprayoon C (2016). The impact of fluid balance on diagnosis, staging and prediction of mortality in critically ill patients with acute kidney injury. Journal of nephrology.

[19] Sime FB, Udy AA, Roberts JA (2015). Augmented renal clearance in critically ill patients: etiology, definition and implications for beta-lactam dose optimization. Curr Opin Pharmacol.

[20] De Waele JJ, Dumoulin A, Janssen A, Hoste EA (2015). Epidemiology of augmented renal clearance in mixed ICU patients. Minerva Anestesiol.

