# Supplementary material for: The Association of Low Admission Serum Creatinine with the Risk of Respiratory Failure Requiring Mechanical Ventilation: A Retrospective Cohort Study
Source: Sci Rep. 2019 Dec 10;9:18743. doi: 10.1038/s41598-019-55362-w (PMC6904463; doi:10.1038/s41598-019-55362-w)
Supplement: Supplementary file 1 — Supplementary information [file 41598_2019_55362_MOESM1_ESM.docx]

Figure S1 Q-Q plot before and after log-transformation for duration of mechanical ventilation in linear regression analysis

1. Q-Q plot before log-transformation


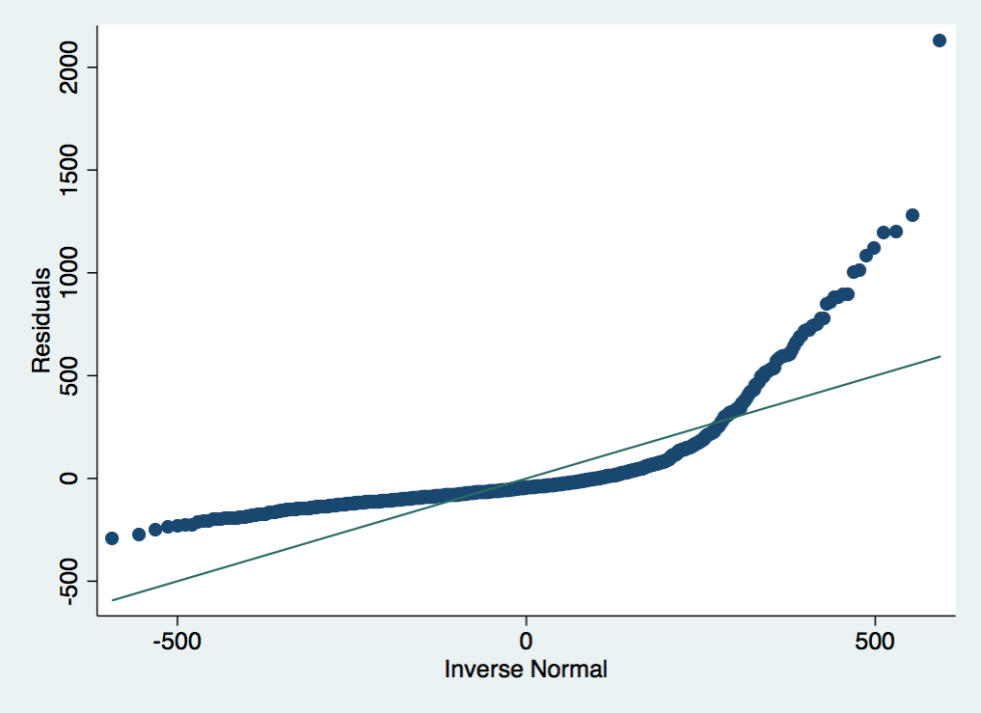


1. Q-Q plot after log-transformation


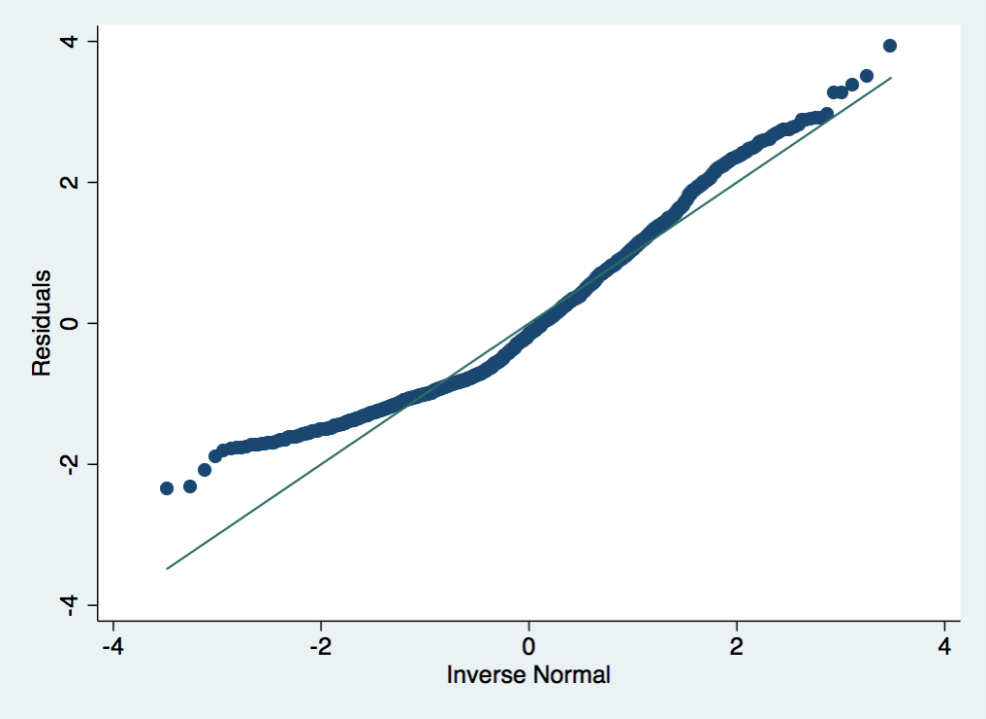


As shown in Q-Q plot, after long-transform the duration of mechanical ventilation, the scatter plot suggested a better normally distributed variable as the residual points follow the straight line. Hence, the normality assumption is held.

Table S1 the association between admission serum creatinine and duration of mechanical ventilation among respiratory failure patients by sex

1. Male

| Serum creatinine level at hospital admission (mg/dl) | Mechanical ventilation duration (hours) | Univariate analysis | | Multivariate analysis | |
| --- | --- | --- | --- | --- | --- |
|  |  | Relative prolongation (95% CI) | p | Relative prolongation (95 % CI)* | P |
| ≤0.4 | 59 (21-141) | 1.33 (0.64-2.73) | 0.44 | 0.81 (0.34-1.92) | 0.63 |
| 0.5-0.6 | 81 (34-135) | 1.55 (1.062.26) | 0.02 | 1.32 (0.89-1.97) | 0.17 |
| 0.7-0.8 | 40 (18-167) | 1.19 (0.93-1.51) | 0.16 | 1.06 (0.83-1.36) | 0.65 |
| 0.9-1.0 | 40 (18-105) | 1 (ref) | - | 1 (ref) | - |
| 1.1-1.2 | 44 (20-97) | 1.09 (0.87-1.38) | 0.44 | 1.13 (0.90-1.43) | 0.29 |
| 1.3-1.4 | 43 (21-117) | 1.10 (0.84-1.44) | 0.50 | 1.14 (0.86-1.50) | 0.37 |
| ≥1.5 | 49 (20-131) | 1.30 (1.03-1.62) | 0.02 | 1.21 (0.95-1.55) | 0.12 |

1. Female

| Serum creatinine level at hospital admission (mg/dl) | Mechanical ventilation duration (hours) | Univariate analysis | | Multivariate analysis | |
| --- | --- | --- | --- | --- | --- |
|  |  | Relative prolongation (95% CI) | p | Relative prolongation (95 % CI)* | P |
| ≤0.4 | 92 (36-305) | 2.08 (1.36-3.16) | 0.001 | 1.81 (1.14-2.85) | 0.01 |
| 0.5-0.6 | 47 (22-158) | 1.35 (1.03-1.77) | 0.03 | 1.34 (1.01-1.79) | 0.04 |
| 0.7-0.8 | 43 (20-86) | 1 (ref) | - | 1 (ref) | - |
| 0.9-1.0 | 43 (17-74) | 0.91 (0.69-1.21) | 0.53 | 0.91 (0.68-1.23) | 0.56 |
| 1.1-1.2 | 44 (22-147) | 1.24 (0.92-1.67) | 0.16 | 1.31 (0.96-1.78) | 0.09 |
| 1.3-1.4 | 67 (22-122) | 1.31 (0.89-1.92) | 0.16 | 1.33 (0.89-1.97) | 0.16 |
| ≥1.5 | 57 (22-119) | 1.26 (0.93-1.70) | 0.12 | 1.34 (0.97-1.86) | 0.08 |

Adjusted for age, race, BMI, principal diagnosis, Charlson Comorbidity Index, coronary artery disease, congestive heart failure, peripheral vascular disease, stroke, diabetes mellitus, chronic obstructive pulmonary disease, cirrhosis, hemi/paraplegia
